# Proposed National Health Board

**Published:** 1879-01

**Authors:** 


### The Proposed National Public Health Board.

Active measures are in progress toward the establishment of a National Public Health Board, for the purpose of improving public hygiene, for carrying out scientific and systematic investigation into the causes and preventive measures of epidemic, contagious and infectious diseases, and in collecting the vital statistics of the United States on some uniform and general plan. A memorial has been drawn up and will be presented to Congress with this object.

It is extraordinary how backward this country is in these matters. The indifference with which all subjects of State medicine are treated by Congress and by State Legislatures would seem to betoken a cynical indifference to human life, health and happiness, on the part of those elected to protect and further these aims. But, in fact, such an interpretation would be an unfair one. The neglect of such subjects arises from ignorance of the vast importance of sanitary science to the welfare of the commonwealth, and extends to the homes and health of the legislators themselves. Last winter we were present one day in the House of Representatives, when one member requested permission of the House to bring before it a matter relating to the heating and ventilation of the chamber, which, he added, was of immediate moment, as involving the health of members. In spite of this, objection was made to the introduction of the subject, and the order of the day was demanded.

This summer has taught a lesson which it seems will bear fruit. The loss by the yellow fever epidemic, one way with another, is set down in round numbers at *two hundred million dollars.* Say that this is a willful exaggeration, and divide it by eight, and there is left a loss in a few months of twenty-five million dollars. Now a most efficient national board of health, with all its officers of able men, with its machinery in good working order, with ample means to print and distribute its results, would cost less than half a million dollars annually. In other words it would cost for fifty years far less than this epidemic has cost in five months! And of its good effect no intelligent man will doubt. We do not hesitate to say that in the future no epidemic of yellow fever equal to that of this summer would ever again visit our land, were such a board organized as it should be, with power to enforce its decisions.

But the Memorial before us asks for no such sum as we have mentioned. Indeed, its request is for a sum so small that any hesitancy in giving it would be inconceivable. It asks for *thirty thousand dollars.* And for what purpose? We quote the proposed organization of the board and the duties it proposes to perform. The provisions are:

I. That the board shall be constituted by one medical officer from each of the following named departments, namely: The medical department of the army, the medical department of the navy. and the marine hospital service, and of two physicians from civil life, selected on account of their knowledge in matters pertaining to public health. That the medical officers of the government services for this board shall be detailed by the secretaries of war, of the navy, and of the treasury respectively, and that the three officers so designated shall select and recommend to the President, for appointment, the other two members of the board.

II. That the board so constituted shall be known as the United States Public Health Board, and that its duties shall be to obtain information upon all matters affecting the public health; to consult with the superintendent of the census with regard to the disease and mortality statistics to be collected and prepared under his direction; to advise the several departments of the government, the executives of the several States, and the commissioners of the District of Columbia, on all questions submitted by them, or whenever in the opinion of the board such advice may tend to the preservation and improvement of the public health. To establish a standard of the qualifications which should be possessed by a municipal health officer, to examine those who may present themselves for examination as to their fitness for such a position, and to furnish suitable formal certificates to those found to be properly qualified.

III. That the detailed officers shall receive their usual payment allowances from their respective departments, and that the two physicians selected from civil life shall each receive compensation at the rate of five thousand dollars per annum from the funds appropriated for the use of this board. That the ordinary sessions of the board shall be in Washington, where it shall have its office, and that at least one member of the board shall be always on duty during the usual office hours.

IV. That the board shall prepare and present to Congress an annual report of its operations, with such recommendations as may seem to it advisable.

V. That when deemed specially desirable by the board it may send one or more of its members to any point within the United States, for the purpose of investigating a disease or other matter especially affecting the public health, and that the actual traveling expenses of the members so sent shall be paid from the funds appropriated for the board.

For the present such an organization is the most that we can hope for. But when this nation deserves to be called an *enlightened* one, when our legislators are awake to their real duties to their constituents, when the government is actualy carried on for the greatest benefit of the people, we shall have a department of the National Government as important as that of War, or for Internal Affairs, with a Secretary of Sanitation in the cabinet, and as a result, epidemic pestilence will be as impossible then as an extensive famine is now.—*Medical and Surgical Reporter.*

